# Targeted mass spectrometry to quantify brain-derived cerebrospinal fluid biomarkers in Alzheimer’s disease

**DOI:** 10.1186/s12014-020-09285-8

**Published:** 2020-05-29

**Authors:** Maotian Zhou, Rafi U. Haque, Eric B. Dammer, Duc M. Duong, Lingyan Ping, Erik C. B. Johnson, James J. Lah, Allan I. Levey, Nicholas T. Seyfried

**Affiliations:** 1 Department of Biochemistry, Emory University School of Medicine, 1510 Clifton Road, Atlanta, GA 30322 USA; 2 Department of Neurology, Emory University School of Medicine, Atlanta, GA 30322 USA

## Abstract

**Introduction:**

Alzheimer’s disease (AD) is the most common cause of dementia, characterized by progressive cognitive decline. Protein biomarkers of AD brain pathology, including β-amyloid and Tau, are reflected in cerebrospinal fluid (CSF), yet the identification of additional biomarkers linked to other brain pathophysiologies remains elusive. We recently reported a multiplex tandem-mass tag (TMT) CSF proteomic analysis of nearly 3000 proteins, following depletion of highly abundant proteins and off-line fractionation, across control and AD cases. Of these, over 500 proteins were significantly increased or decreased in AD, including markers reflecting diverse biological functions in brain. Here, we use a targeted mass spectrometry (MS) approach, termed parallel reaction monitoring (PRM), to quantify select CSF biomarkers without pre-depletion or fractionation to assess the reproducibility of our findings and the specificity of changes for AD versus other causes of cognitive impairment.

**Method:**

We nominated 41 proteins (94 peptides) from the TMT CSF discovery dataset, representing a variety of brain cell-types and biological functions, for label-free PRM analysis in a replication cohort of 88 individuals that included 20 normal controls, 37 clinically diagnosed AD cases and 31 cases with non-AD cognitive impairment. To control for technical variables, isotopically labeled synthetic heavy peptide standards were added into each of the 88 CSF tryptic digests. Furthermore, a peptide pool, representing an equivalent amount of peptide from all samples, was analyzed (*n *= 10) across each batch. Together, this approach enabled us to assess both the intra- and inter-sample differences in peptide signal response and retention time.

**Results:**

Despite differences in sample preparation, quantitative MS approaches and patient samples, 25 proteins, including Tau, had a consistent and significant change in AD in both the discovery and replication cohorts. Validated CSF markers with low coefficient of variation included the protein products for neuronal/synaptic (GDA, GAP43, SYN1, BASP1, YWHAB, YWHAZ, UCHL1, STMN1 and MAP1B), glial/inflammation (SMOC1, ITGAM, CHI3L1, SPP1, and CHIT1) and metabolic (PKM, ALDOA and FABP3) related genes. Logistical regression analyses revealed several proteins with high sensitivity and specificity for classifying AD cases from controls and other non-AD dementias. SMOC1, YWHAZ, ALDOA and MAP1B emerged as biomarker candidates that could best discriminate between individuals with AD and non-AD cognitive impairment as well as Tau/β-amyloid ratio. Notably, SMOC1 levels in postmortem brain are highly correlated with AD pathology even in the preclinical stage of disease, indicating that CSF SMOC1 levels reflect underlying brain pathology specific for AD.

**Conclusion:**

Collectively these findings highlight the utility of targeted MS approaches to quantify biomarkers associated with AD that could be used for monitoring disease progression, stratifying patients for clinical trials and measuring therapeutic response.

## Introduction

Alzheimer’s disease (AD) alone afflicts roughly 5.4 million individuals in the United States and 24 million worldwide, and the prevalence is increasing with longer lifespans and the absence of effective disease-modifying therapies [1]. Currently, positron-emission tomography (PET) imaging and cerebrospinal fluid (CSF) measures of β-Amyloid (Aβ) and Tau allow identification of pre-symptomatic individuals at risk of AD, improve diagnostic accuracy for symptomatic individuals, and help to stratify appropriate subjects for clinical trials [2, 3]. However, the failures of several clinical trials of Aβ-based therapeutic approaches highlight the need for a fuller understanding of AD as a complex disease involving mechanisms beyond Aβ and Tau deposition in brain [4]. For example, most cases of dementia are due to a complex mixture of pathologies that are also seen in aging and other neurodegenerative diseases [5]. These pathological phenotypes extend beyond the hallmark protein aggregates that define these diseases to synapse loss, inflammation, metabolism and other cellular, molecular and biochemical changes that are now being appreciated as key pathophysiological mechanisms and therapeutic targets [6–10]. Thus, there is a need to identify additional biomarkers that reflect underlying brain processes in AD and related disorders [2]. Ultimately these biomarkers could be used to stage disease progression, identify patients for clinical trials and assess target engagement of novel AD therapeutics [11].

CSF has become one of the most promising sources for accessible biomarkers of neurodegenerative disease as it maintains direct contact with the brain and therefore may reflect biochemical changes co-occurring with AD neuropathology [12]. Advances in liquid chromatography coupled to tandem mass spectrometry (LC–MS/MS) now facilitate high-throughput detection and quantification of proteins in complex mixtures including CSF [13–15]. Furthermore, coupling off-line fractionation and multiplex isobaric labeling using tandem mass tags (TMT) enables the precise quantitation of thousands of proteins across many samples simultaneously for large-scale discovery proteomic applications [16, 17]. To this end, we recently reported a TMT based CSF proteomic analysis of ~ 3000 proteins across 20 controls and 20 AD patients [6]. Of these, over 500 proteins were significantly increased or decreased in the CSF of AD patients, including markers reflecting diverse biological functions with strong correlation with Aβ and Tau CSF levels.

Here we nominated 41 proteins, including Tau, from this deep CSF discovery dataset [6], representing a variety of specific cell-types and biological functions, for validation using a targeted proteomics approach termed parallel reaction monitoring (PRM) in a separate cohort of 88 individual CSF samples. PRM combines the high sensitivity and multiplex ability of targeted-MS experiments, such as selected reaction monitoring (SRM), together with the specificity of high-resolution MS [18]. This replication cohort included 20 normal controls, 37 clinically diagnosed AD cases and 31 cases with non-AD cognitive impairment. Cases were recruited from a neurology specialty clinic to mirror real world application and performance in a group of symptomatic individuals reflecting a spectrum of AD and non-AD etiologies for cognitive impairment. Collectively, 25 proteins were significantly and specifically increased in AD compared to controls and other non-AD cases. Logistic regression and receiver operator characteristic (ROC) analyses revealed several proteins with excellent sensitivity and specificity in classifying AD diagnosis and biomarker status. Namely, SMOC1, ALDOA, MAP1B and YWHAZ proteins emerged as biomarker candidates with low coefficients of variation that could best discriminate AD from non-AD cases with cognitive impairment as well as predict individuals with high Tau/Aβ ratio in CSF. Notably, SMOC1 levels in postmortem brain were also highly correlated with AD pathology even in the preclinical stage of disease indicating that CSF SMOC1 levels reflect underlying brain pathology specific for AD. Collectively, these findings highlight the utility of MS-based proteomics to identify biomarkers associated with AD that could be used for monitoring disease progression, stratifying patients for clinical trials and measuring therapeutic response.

## Materials and methods

### Materials

Trypsin, mass spectrometry grade was bought from ThermoFisher Scientific (Waltham, MA). Lysyl endopeptidase (Lys-C), mass spectrometry grade was bought from Wako (Japan); CAA (chloroacetamide), TCEP (tris-2(-carboxyethyl)-phosphine), and triethylammonium hydrogen carbonate buffer (TEAB) (1 M, pH 8.5) were obtained from Sigma (St. Louis, MO). Heavy labeled AQUA™ Peptides were purchased from ThermoFisher Scientific (Waltham, MA). Notably, the HPLC purity was determined to be > 95% via quantification performed by amino acid analysis. Glass inserts for liquid chromatography auto-sampler were from Wheaton (Millville, NJ).

### Human CSF collection and immunoassays

All symptomatic individuals were diagnosed by expert clinicians in the ADRC and Emory Cognitive Neurology, who are subspecialty trained in Cognitive and Behavioral Neurology, following extensive clinical evaluations including detailed cognitive testing, neuroimaging, and laboratory studies. CSF samples from 88 individuals in the replication cohort included 20 healthy controls, 37 patients with mild, symptomatic AD (either prodromal AD with mild cognitive impairment or early stage AD dementia with positive AD biomarkers), and 31 with other (non-AD) neurological disease (Additional file 1: Tables S1 and S2). The three groups were matched as closely as possible for age and sex. For clinical testing, CSF samples from these individuals were either sent to Athena Diagnostics and assayed for Aβ42, total-Tau, and phospho-Tau (CSF ADmark^®^) using the INNOTEST^®^ assay platform or sent to Akesogen (Norcross GA) and assayed for Aβ42, total-Tau, and phospho-Tau using the Multiplex xMAP technology (Luminex Corporation). Although these two assay platforms yield different absolute values for Aβ42, total-Tau, and phospho-Tau, the relative values are highly correlated as previously described [19].

### Protein digestion of CSF

CSF samples for PRM analyses were prepared essentially as described [20]. Samples were thawed at room temperature and protein concentrations measured by the bicinchoninic acid assay (BCA) method (Additional file 1: Table S2). CSF (20 µL) and each sample was reduced and alkylated by adding 5 μL of 50 mM TCEP, 200 mM CAA, 250 mM ammonium bicarbonate, pH 8.0. Each sample was vortexed for 30 s and then heated at 90 °C for 10 min followed by bath sonication for 10 min. Isotopically heavy labeled peptide standards (7 peptides total) were diluted with water from a stock solution (5 pmol/μL, 5% ACN in water) to working solution (10×). From the stock solution, 2µL of each peptide were added to the digestion solution (equal to CSF volume). Digest solution (50 µL of Lys-C enzyme/protein ratio 1:10 in 8 M of urea) was added to the CSF samples and digested overnight. The samples were then diluted with 50 mM ammonium biocarbonate buffer (1:6 (v/v)), and trypsin was added at enzyme/protein ratio 1:10 (w/w). Samples were further digested at room temperature overnight with agitation. After the incubation, the samples were quenched by adding trifluoroacetic acid and formic acid (final concentration, 0.1% TFA, 1% FA). The samples were desalted using 50 mg tC18 columns (Waters, Milford, MA) according to the manufacturer’s protocol and eluates were dried under vacuum.

### Quality control analyses using internal and external reference standards

Prior to mass spectrometry analyses, the 88 CSF sample digests were randomized and divided into 4 batches (Additional file 2: Fig. S1). To control for technical variables, isotopically labeled synthetic heavy peptide standards (Promega 6 × 5 LC–MS/MS Peptide Reference Mix) were added into each of the 88 CSF tryptic digests. Furthermore, a peptide pool, representing an equivalent amount of peptide from all 88 samples, was analyzed at different injection positions across the 4 batches as previously described [20]. Together, this approach enabled us to assess both the intra- and inter-sample differences in signal response and retention time, which allowed us to normalize for peptide signal drift across batches. We selected 94 peptides across each of the 41 targets proteins based on empirically generated proteomics data from control and AD CSF patients [6] (Additional file 2: Fig. S1), which increases the likelihood for success in developing a targeted MS-based assay [21]. Peptides specific to the APOE2 and APOE4 allelic variants were also targeted, which allowed us to confirm person-specific APOE genotype. Finally, to assess the accuracy of the label-free PRM measurements heavy labeled isotopic standards (AQUA™ Pro, ThermoFisher) were included for a subset of peptides and the light/heavy ratios compared to the normalized peak area (Additional file 2: Fig. S2).

### Parallel reaction monitoring (PRM) analysis

Each sample (equal to 2 µl CSF) was analyzed on a Q-Exactive Plus hybrid mass spectrometer (ThermoFisher Scientific) fitted with a Nanospray Flex ion source and coupled to a NanoAcquity liquid chromatography system (Waters Corporation) essentially as described [20]. The tryptic peptides were resuspended in 40 μL of loading buffer (2% ACN, 0.1% TFA) and 2 µl was loaded onto a self-packed 1.9 um ReproSil-Pur C18 (Dr. Maisch) analytical column (New Objective, 30 cm × 75 µm inner diameter; 360 µm outer diameter). Elution was performed over a 40-min gradient at a rate of 300 nL/min with buffer B ranging from 2 to 25% (buffer A: 0.1% formic acid in water, buffer B: 0.1% formic acid in ACN). The column was then washed with 99% B for 40 min and re-equilibrated with 2% B for 15 min. The mass spectrometer was set to collect in PRM mode with an inclusion list consisting of each peptide (Additional file 1: Table S3). For PRM scans, the settings were: resolution of 35,000 at 200 m/z, AGC target of 5 × 10^5^ ions, max injection time of 200 ms, loop count of 30, MSX count of 1, isolation width of 1.6 m/z and isolation offset of 0.5 m/z. A pre-optimized normalized collision energy of 28% was used to obtain the maximal recovery of target product ions. A series of product ions from this collision energy optimization were used for downstream peptide quantification.

### Peptide quantification

An in-house spectral library was built using Skyline [22] (Version 4.2) based on tandem mass spectra gathered from CSF following high pH fractionation. A Skyline template was created to quantify the peptides. The template parameters were: Precursor mass analyzer, Centroided; MS1 mass accuracy of 20 ppm; Product mass analyzer, Centroided; MS/MS Mass accuracy of 20 ppm; include all matching scans. All rawfiles were then imported and processed accordingly. The resulting extracted ion chromatograms (XICs) of selected fragments were manually inspected and peak picking adjustments were made accordingly. The sum of all product ion peak areas was calculated by Skyline and extracted for further statistical analysis. By matching from the spectrum library, the average dot product ions (Dotp) value for all the peptides was 0.87. The summed product ions peak areas were considered as raw peptide peak area (Additional file 1: Table S3), which was normalized by external reference heavy peptide standards to correct for MS signal and sample loading variance (Additional file 1: Table S4). The sum of all peptides belonging to the same protein was calculated and normalized by the average of global pooled (GIS) samples (Additional file 1: Table S5). Protein normalized ratios were then regressed by age and gender, and centered to mean zero (Additional file 1: Table S6) prior to statistical analyses.

### Statistical analysis

Statistical analyses were performed in GraphPad Prism version 7.00 for Windows (GraphPad Software, San Diego California USA). For endogenous peptides having corresponding heavy peptides, the light to heavy ratios were directly calculated by dividing the endogenous peptide peak area by the corresponding heavy peptide peak area. For proteins, standard Student’s t-test, or one-way ANOVA test were used to calculate significance (Additional file 1: Table S7).

### Correlation analyses

In the discovery TMT dataset, the Bicor rho correlation, and Student’s significance p values, were calculated using the WGCNA R package using the bicor And P value function, comparing normalized protein sum of peptide measurements by MS to selected traits obtained on paired cases by ELISA measurements of total Tau, phospho-Tau (pT181), β-amyloid, and ratio of Tau/Aβ (Additional file 1: Table S8). Only samples assayed on the Luminex technology (Akesogen) were considered in the analyses. A second outlier was also not included due to high human serum albumin levels as previously reported [6].

### ROC analyses

For each protein, a logistic regression classifier (MATLAB ver. R2018b, Natick, Massachusetts USA) was trained to classify individuals as healthy control or AD dementia based on clinical diagnosis. In a similar manner, logistic regression classifiers were trained to classify individuals with AD dementia or non-AD cognitive impairment. The last logistic regression classifier was trained to classify a biological profile suggestive of AD using an amyloid/tau ratio CSF cutoff. A total of 67 samples (Akesogen) were included in the Aβ/Tau ratio analyses using a ratio threshold for AD biomarker positivity of 4.6 as previously reported [23]. These three logistic regression classifiers for each protein were trained using a five-fold cross validation procedure and the performance of these classifiers was assessed on the testing set using the AUC of the ROC curve (Additional file 1: Table S9).

## Results

### Prioritization of AD CSF biomarkers for targeted mass spectrometry

**Fig. 1:**
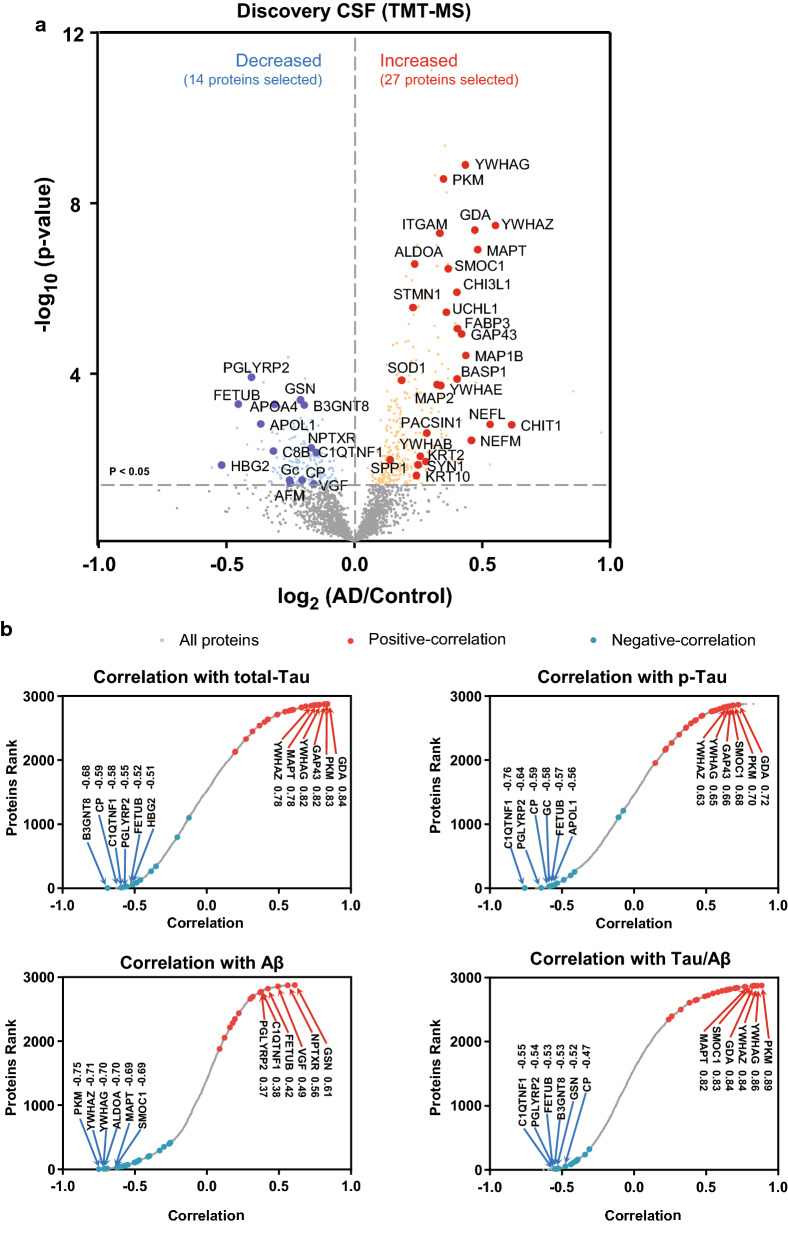
Nominated candidate targets and their correlation to AD biomarkers. **a** Volcano plot displaying the log_2_ fold-change (x-axis) against significance,  −log_10_ p value, for all proteins (n = 2875), including those differentially expressed between control and AD cases of the TMT discovery. Proteins above the dashed line (> 1.3) on the y-axis are statistically significant (p < 0.05). Proteins that are depicted in red (increased) or blue (decreased) denote the 41 significantly different proteins quantified by PRM in this study. **b** Correlation of all proteins quantified in the TMT discovery cohort (n = 2785) with paired ELISA measures of t-tau, pTau, Aβ, and Aβ/Tau ratio. Proteins highlighted in red or blue reflect the 41 targets either positively or negatively correlated to the ELISA values. All protein correlations and p-values are provided in Additional file 1: Table S8

We recently reported a discovery proteomic analysis (~ 3000 proteins) of CSF from control and AD cases (*n *= 40) using multiplex isobaric tandem mass tags (TMT) [6]. To assess the reproducibility of our findings and the specificity of the changes for AD versus other causes of dementias, we used a targeted mass spectrometry approach, parallel reaction monitoring (PRM), for analysis of a second replication cohort of patients that included 20 cognitively normal controls, 37 AD cases and 31 cases with non-AD cognitive impairment or dementia. The latter group were cognitively impaired, but negative for Aβ and Tau biomarker status (Additional file 1: Table S1 and S2). Unlike the discovery dataset, the depletion of highly abundant proteins and pre-fractionation was not performed prior to PRM in the replication cohort. This ultimately restricted our analyses to proteins which had detectable peptides in un-depleted CSF in a ‘single-shot’ LC–MS/MS assay. Thus, we focused our PRM analyses on 41 proteins that were significantly changed (increased or decreased) in AD CSF of the discovery case samples (Fig. 1a). These included several previously reported AD biomarkers [24, 25], such as microtubule-associated protein tau (MAPT) [26], neurofilament light chain protein (NEFL) [27], growth associated protein 43 (GAP43) [28], fatty acid binding protein 3 (FABP3) [29], osteopontin (SPP1) [30, 31], and chitinase 3 like 1 (CHI3L1; also known as YKL-40) [32]. Nearly all of the 41 proteins were significantly correlated with Aβ, Tau or phosphorylated Tau (pTau) ELISA levels in the discovery cohort (Fig. 1b and Additional file 1: Table S8).

### Quantification of CSF biomarkers using targeted mass spectrometry

We selected 94 tryptic peptides from the 41 target proteins based on empirically generated proteomics data from control and AD CSF cases [6]. The technical coefficient of variation (CV) of each peptide was calculated based on a pooled standard of all samples (*n* = 10 replicate injections) following batch normalization (Additional file 2: Fig. S1A and B), in which the average CV for all peptides targeted was ~ 27%. All targeted peptides and CVs following batch normalization are provided in Additional file 1: Table S4. Peptide measurements were summed for proteins with more than one peptide quantified to generate a single protein measurement (Additional file 1: Table S5). After summing the values for a protein measurement, the average CV for each protein was ~ 25% (Additional file 2: Fig. S1C), which is consistent with the technical CVs reported for label-free proteomic assays [33, 34]. Thus, we defined CSF biomarkers with CVs above and below 30% as quantified with low and high precision, respectively. In total, 71% of all proteins quantified were quantified with high precision. Thus, these data support the utility of label-free PRM mass spectrometry to detect robust AD biomarkers with good precision in un-depleted and unfractionated CSF samples.

### Tau peptides quantified by PRM correlate to Tau measured by immunoassays

**Fig. 2:**
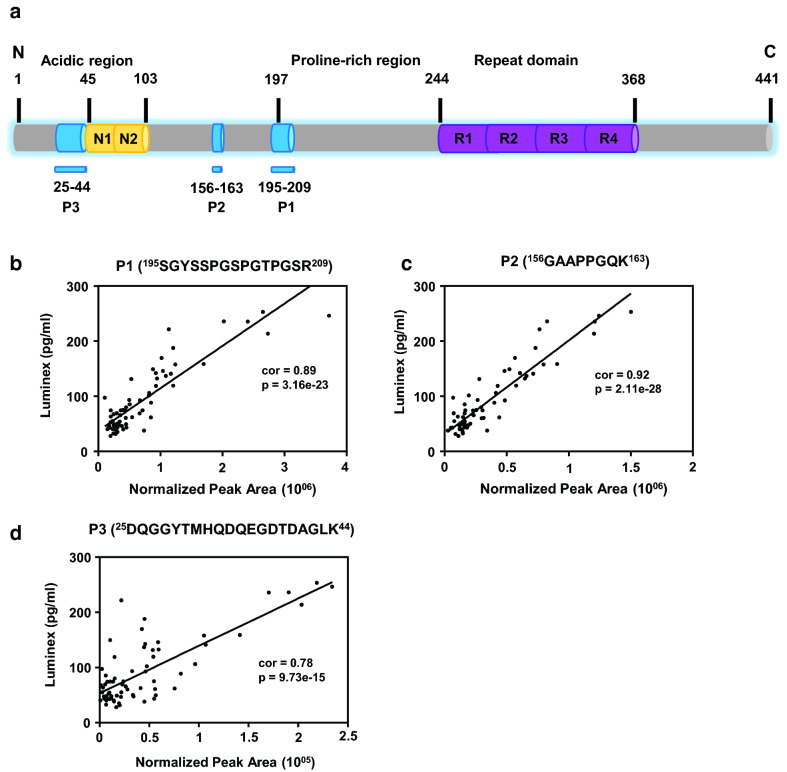
Tau peptides quantified by PRM correlate to Tau measurements by immunoassay. **a** Three Tau peptides mapping to the mid-domain (P1 and P2) or the N-terminal acidic region (P3) were quantified by label-free PRM in the replication cohort. The normalized peak area measurements for P1, P2, and P3 were compared with total Tau level measurements by immunoassay in a subset of samples (*n *= 67). **b** P1, mapping to residues 195-209 of Tau, showed strong correlation (cor = 0.89, p = 3.16e−23) with Tau levels by Luminex immunoassays. **c** Label free peak area for Tau P2, mapping to residues 156–163, was also significantly correlated to Tau immunoassay (cor = 0.92, p = 2.11e−28). **d** Label-free peak area of P3, mapping to residues 25–44, also correlated to immunoassay measurements (cor = 0.78, p = 9.73e−15)

The predominant form of tau in CSF contains the proline-rich mid-domain (residues 103–204) and, for the most part, lacks the microtubule binding repeat and C-terminus peptides [35] (Fig. 2). Thus, we targeted Tau peptides P1 and P2 mapping to the mid-domain, or P3 in the N-terminal acidic region and compared these measurements to total Tau levels by immunoassays in a subset of samples measured using xMAP technology (*n *= 67). Consistent with our previous findings [20] and other reports [35, 36], Tau P1, mapping to residues 195–209, showed strong correlation (cor = 0.89 p = 3.16e−23) with Tau levels by ELISA [37, 38]. Similarly, a second Tau peptide (P2), mapping to the mid-domain region (residues 156–163), was also significantly correlated to Tau ELISA values (cor = 0.92, p = 5.42e^−33^). In contrast, the P3 tau peptide (residues 25-44) was less correlated to immunoassay measurements (cor = 0.78), yet still highly significant (p = 9.73e−15). These results indicate that label-free PRM quantification results for Tau levels for all three N-terminal peptides are highly consistent with total Tau levels measured by ELISA.

### AD specific CSF biomarkers validate in replication cohort

**Fig. 3:**
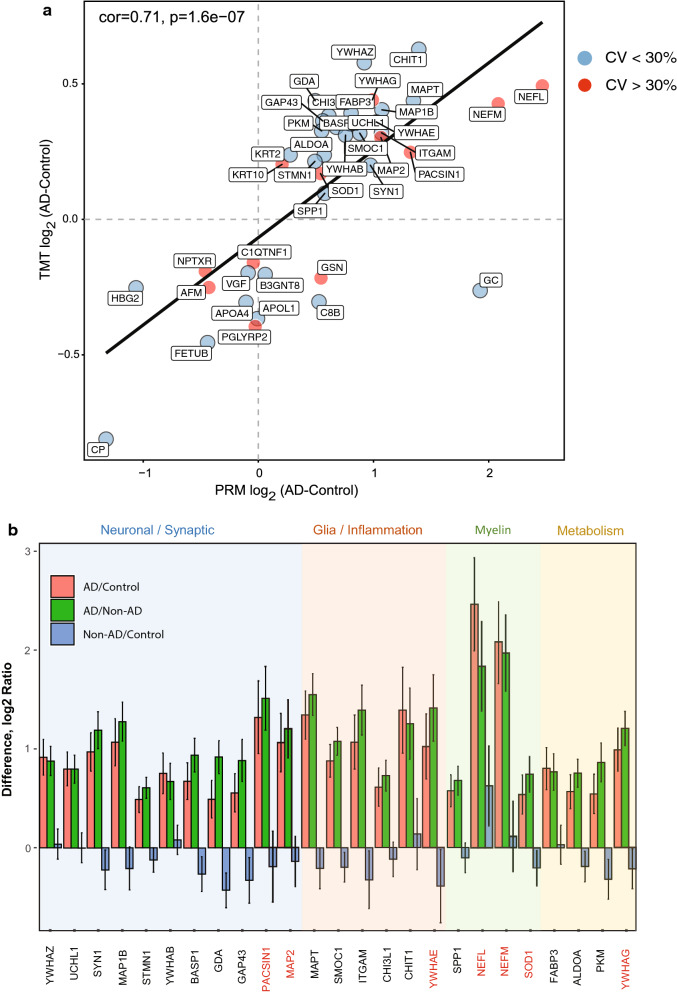
AD specific CSF markers reflect underlying changes in synaptic transmission, inflammation, myelination and energy metabolism. **a** Linear regression analyses showed a strong degree of correlation (cor = 0.71, p = 1.6E−7) between the 41 targeted proteins in AD CSF compared to control in both the discovery (TMT-MS) and replication (PRM) cohorts despite difference in sample preparation and quantitative MS platforms. Proteins with high CV (> 30%) and low CV (< 30%) are represented by red and blue circles, respectively. **b** Following ANOVA analyses (Tukey post hoc FDR), a total of 25 of 41 proteins (61%) were consistently and significantly increased in AD (p < 0.05) compared to controls and non-AD dementia cases. Proteins are classified by their biological and/or cell type expression profiles in brain (background color shading) as neuronal/synaptic (blue), glial/inflammation (orange), myelin (green) and metabolism (yellow). Proteins highlighted in red have a CV > 30%. Error bars represent standard error of the mean

As noted above, there are differences in how the CSF proteins were prepared and quantified across the discovery and replication cohorts. Thus, we first assessed whether the 41 nominated biomarkers from the discovery cohort had a consistent direction of change in AD in the replication cohort (Fig. 3a). Linear regression analysis showed a strong degree of correlation (cor = 0.71, p = 1.6e^−7^) between the 41 targeted proteins in both the discovery and replication cohorts. The majority of the proteins that replicated in the discovery cohort were increased in AD CSF compared to controls (Figs. 1 and 3a). Exceptions included GSN, C8B and GC, which were decreased in AD within the discovery cohort, yet increased in AD in the replication cohort. A total of 25 out of 41 proteins (61%) were also consistently and significantly increased in AD across both cohorts. This included Tau (MAPT) and neurofilament light and medium chain proteins (NEFL and NEFM) (Fig. 3b and Additional file 2: Figure S3). All the proteins that discriminated AD from normal controls were also significantly increased in AD compared to patients with non-AD dementia. However, higher levels of NEFL were observed in patients with non-AD dementia, compared to controls, consistent with previous studies [39]. Thus, we conclude that these 25 replicated biomarker measurements are robust, regardless of the substantial differences in MS instrumentation and protein quantitation approaches, and they are not influenced by whether the samples are pre-depleted for highly abundant proteins.

### Validated CSF markers reflect underlying changes in synaptic transmission, inflammation, myelination and energy metabolism in AD brain

We recently implemented an integrated systems-based approach to identify brain-derived CSF biomarker panels that reflect a range of disease physiology [6]. Using this approach, we classified CSF biomarkers into five panels that mapped broadly to biochemical and cellular phenotypes in the AD brain, including synaptic transmission, vascular biology, myelination, glial-mediated inflammation, and energy metabolism [6]. Notably, 26 of the 41 CSF markers quantified in this study, mapped to one of these five biomarker panels [6] (Fig. 3b, Additional file 2: Fig. S3 and Additional file 1: Table S7). Overlap with the neuronal/synaptic panel included the protein products of 11 genes (MAP2, GDA, NPTXR, GAP43, VGF, SYN1, BASP1, PACSIN1, YWHAB, YWHAZ, and UCHL1) and with the exception of NPTXR and VGF, which decreased in the discovery CSF dataset, all other neuronal panel members were significantly and specifically increased in the AD replication cohort. The increase in synaptic CSF markers in the replication cohort is also consistent with the divergent brain-CSF expression trends displayed for the synaptic markers observed in our previous study, in which a majority of synaptic proteins in CSF were observed to have increased levels, yet predominantly display decreased levels in the AD brain [6]. Glia/inflammation panel members SMOC1, ITGAM, and MAPT also replicated in AD CSF [6], and with the exception of GSN and KRT2, all overlapping members of the myelin panel (NEFL, NEFM, SPP1, and SOD1) and metabolism panel (PKM, FABP3, and ALDOA) were also consistent between discovery and replication cohorts (Fig. 3b). Only two members of the vascular panel (C8B and CP) were targeted and, although ceruloplasmin (CP) was decreased in AD, neither target met significance in the replication cohort. Additional significantly increased AD markers that fell outside the five brain-derived CSF panels included YWHAE, CHI3L1, CHIT1, MAP1B and STMN1. Based on their cell-type RNA expression [40, 41] and human brain protein co-expression profiles [6, 8] we assigned STMN1 and MAP1B to the synaptic transmission panel and YWHAE and CHI3L1 to the glial-mediated inflammation panel (Fig. 3b). Although low levels of CHIT1 transcript and protein levels are detected in brain [40, 41], this CSF marker has been previously linked to neuroinflammation [42, 43] and therefore, was assigned to the glial-mediated inflammation panel (Fig. 3b).

### Protein classifiers predict clinical diagnosis and β-amyloid/Tau biomarker ratio of AD

**Fig. 4:**
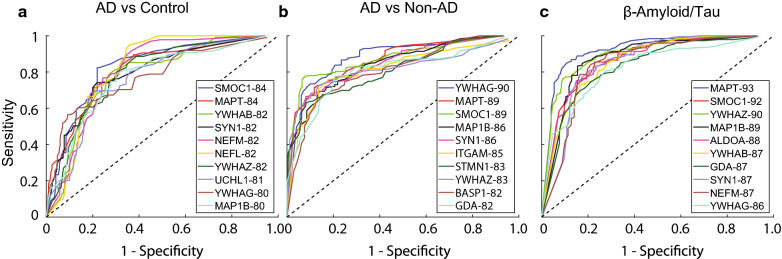
Logistic regression analysis prioritizes CSF protein biomarkers that best classify AD. Logistic regression followed by ROC analysis for each of the 41 biomarkers alone was assessed. The top performing protein markers that classified AD from control (**a**) or AD from non-AD cases (**b**) are highlighted. **c** ROC curves for top performing proteins that predict classification of cases based on Aβ/Tau ratio. All protein AUCs are provided in Additional file 1: Table S9.

To assess the degree to which any of these CSF proteins could predict clinical diagnosis, a logistic regression classifier was employed (Fig. 4 and Additional file 1: Table S9). The performance of these classifiers was assessed using the area under the curve (AUC) of the receiver operating characteristic (ROC) curve in which the AUC provides a measure of the overall performance of a diagnostic test with a value between 0 and 1 (Fig. 4). The top protein classifiers for AD versus control clinical diagnosis included members of the glia/inflammation panel, SMOC1 and MAPT (AUC = 0.84), followed by the synaptic 14-3-3 protein YWHAB (AUC = 0.82). MAPT and SMOC1 were also two of the top predictive markers for AD compared to non-AD patients (AUC = 0.89), although both slightly trailed YWHAG (0.90). Finally, we assessed which protein(s) could predict the ratio of Aβ/Tau levels measured by immunoassay (*n *= 67). MAPT had the highest AUC at 0.93, as expected, followed by SMOC1 (0.92), YWHAZ (0.90), MAP1B (0.89) and ALDOA (0.88). The ability of these proteins to predict Aβ/Tau ratios is consistent with SMOC1, ADLOA and YWHAZ being highly correlated to Tau/Aβ levels in the discovery dataset (Fig. 1b).

## Discussion

In this study, over 40 AD associated CSF proteins, including Tau, were quantified using a targeted proteomics approach in a cohort that included patients with non-AD cognitive impairment. Despite differences in sample preparation, quantitative MS approaches and patient samples, 25 proteins, including Tau and neurofilaments, were found significantly increased in AD compared to controls and patients with non-AD cognitive impairment. Furthermore, logistic regression revealed several proteins with high predictive power for classifying AD cases. Proteins quantified with high precision that emerged from these analyses included YWHAZ, ALDOA and SMOC1, which map to synaptic, metabolism and neuroinflammation pathways, respectively, in brain [6, 8]. These findings begin to prioritize biomarkers associated with diverse AD pathophysiologies that could be used for classifying patient populations and possibly for monitoring disease progression and therapeutic response.

One goal of our study was to determine whether specific peptides representing candidate biomarkers in AD CSF could be reliably quantified in a “single-shot” targeted mass spectrometry assay without depletion or fractionation. To achieve this goal, we employed a label-free PRM approach, which provides an expedited assessment of the reproducibility and detection of peptide biomarkers in CSF. However, one limitation of label-free approaches is the high CV for certain targeted peptides, which can be reflective of the overall lower levels of these peptides in the sample. However, in this study, the majority (> 70%) of the targeted peptides and proteins quantified had CVs < 30%, indicating they were reliably detected in human CSF without depletion. Thus, higher CVs for the minority of proteins quantified in this study likely reflect their low abundance in CSF. In particular, the neurofilament proteins (NEFL and NEFM) had high CVs and were near the lower limit of detection by PRM in the pooled standards. Our findings are consistent with a previous PRM study for NEFM, in which it was detected in certain CSF pools, but not following dilution [39]. Despite the low levels of NEFL and NEFM in the CSF pooled standards used in this study, we confirmed the identity of both peptides by MS/MS fragmentation using synthetic peptide standards (Fig. S4) and elected to include these targets in the analyses given the large effect size for both proteins. Ultimately, as candidate proteins and corresponding peptides with low CVs are promoted from label-free PRM assays into selected reaction monitoring (SRM) assays on triple quadruple mass spectrometers, the lower limit of quantification (LLOQ) and linearity of quantification will need to be determined using a dilution series in human CSF with pure isotope labeled heavy standard peptide, as we have described previously for Tau [20]. These standardized assays will be vital for the successful development and deployment of clinical assays in a CLIA-regulated environment [21].

A second goal of this study was to assess whether the targeted AD candidate biomarkers were consistent in their direction of change in AD CSF when measured in a non-depleted sample. To this end, we observed strong concordance between the direction of change between the proteins in the discovery and replication cohorts despite differences in sample preparation and mass spectrometry platforms. Indeed, some of the most differentially increased proteins in the discovery CSF dataset (SMOC1, MAPT, YWHAG, and PKM), were highly correlated across the discovery and validation datasets. It should be noted that GSN, C8B, and GC were decreased in AD within the discovery cohort, yet increased in AD in the replication cohort. This difference could be due to the distinct sample preparation procedures between the discovery and validation cohort. For example, the protein depletion procedure used in the discovery cohort can influence levels of specific proteins that interact with the highly abundant proteins depleted in the samples [44]. All of the 25 significantly higher proteins in AD compared to controls were also specific to AD when compared to patients with non-AD cognitive impairment. This included several synaptic proteins in CSF, which may reflect AD-specific changes linked to dendritic spine structure and synaptic remodeling due to high Aβ burden in brain [45, 46]. Notably, synaptic loss correlates more closely with cognitive dysfunction than Aβ and tau pathologies, indicating that these proteins reflecting synaptic dysfunction could also be excellent therapeutic targets and prognostic biomarkers of disease [47, 48]. Despite the specificity observed for these proteins in AD, it should be noted that it is unclear the extent of neurodegeneration or cause of dementia in the non-AD individuals. Thus, our findings support the need for further assessment of the sensitivity and specificity of the biomarkers for AD compared to other neurodegenerative diseases (e.g. Parkinson’s disease, frontotemporal dementia, amyotrophic lateral sclerosis, etc.) with definable clinical and pathological phenotypes affecting differential secretion into CSF.

Based on their correlation to AD biomarkers (Aβ and Tau) and sensitivity and specificity of classifying AD cases by ROC analyses, several biomarkers emerged from this study that may be related to underlying AD brain pathology. Namely, SMOC1 levels in postmortem brain were highly correlated with AD pathology even in the preclinical stage of disease [6, 7, 49] indicating that CSF SMOC1 levels reflect underlying brain pathology. SMOC1 is a secreted modular calcium-binding protein, which is localized within the basement membrane in kidney and skeletal muscle [50], and has a critical role in ocular and limb development [51]. Previous studies indicate SMOC1 is an ALK5 antagonist produced by endothelial cells that drives TGF-β signaling towards ALK1 activation, thus promoting endothelial cell proliferation and angiogenesis [52]. Furthermore, SMOC1 levels are detectable in plasma and are associated with aging [53]. Thus, future studies that assess SMOC1 plasma levels in AD patients are warranted. Other notable targets that were increased in AD included the 14-3-3 proteins, YWHAZ, YWHAG and YWHAB. These proteins were originally discovered as a family of proteins that are highly expressed in the brain [54]. Through interactions with a multitude of binding partners, 14-3-3 proteins impact many aspects of brain function including neural signaling, neuronal development and neuroprotection and have been previously linked to AD and Creutzfeldt-Jakob Disease (CJD) in CSF [54]. Although we see specificity for AD with 14-3-3 proteins compared to patients with non-AD cognitive impairment, it is unclear the extent of neurodegeneration in the non-AD individuals. Fructose-bisphosphate aldolase A (ALDOA) from the energy metabolism panel emerged as a target that can classify AD from non-AD patients. ALDOA is a glycolytic enzyme that catalyzes the reversible conversion of fructose-1,6-bisphosphate to glyceraldehyde 3-phosphate and dihydroxyacetone phosphate, and heightened levels of ALDOA likely reflect the increased levels of sugar/glycolytic metabolism in brain [7, 10].

## Conclusions

Collectively, these findings highlight the utility of MS-based proteomics to identify biomarkers associated with AD beyond Aβ and Tau. Future studies assessing the longitudinal profiles of these biomarkers will be critical to determine which combination of markers best correlate with disease severity. Ultimately, the accurate and robust measurement of biomarkers from this study across additional AD cohorts could also potentially help stratify individuals at risk for developing AD and subsequent enrollment into clinical trials.

## Supplementary information

**Additional file 1: Table S1.** Summary of replication cohort characteristics. **Table S2.** Clinical and immunoassay metadata for all 88 cases. **Table S3.** Raw PRM quantified peptide peak area before normalization. **Table S4.** Normalized peptide peak areas. **Table S5.** Normalized Protein Quantification. **Table S6.** Normalized Protein Quantification (Regressed for age and sex). **Table S7.** Differential Expression Analysis (ANOVA). **Table S8.** Correlation of all proteins quantified in the TMT discovery cohort with ELISA-measured t-tau, pTau, Aβ, and Aβ/Tau ratio. **Table S9.** ROC analysis.

**Additional file 2: Figure S1.** Overview of PRM targeted experiment. **(A)** Schematic of the workflow of the targeted PRM analyses of the replication cohort. Samples (n = 98 total) including 37 AD, 20 Controls, 31 non-AD dementia cases, and 10 pooled global internal standards (GIS) were randomized (e.g. diagnosis, age and sex) into four batches. **(B)** The raw and normalized peak areas (based on intensities) of reference peptides LASVSVSR, LASVSVSR, YVYVADVAAK, YVYVADVAAK, VVGGLVALR, VVGGLVALR, LLSLGAGEFK, and LLSLGAGEFK (heavy amino acids were labeled with underline) across the first and second injection of 98 samples (including 10 GIS samples). **(C)** The left panel shows the histogram for the coefficient of variation (CV) of all peptides quantified across the 10 GIS samples before normalization with an average coefficient of variation of 44%. The middle panel showing the coefficient of variation of all peptides quantification across 10 GIS samples after normalization with an average coefficient of variation of 27%. The right panel shows the coefficient of variation of 41 proteins quantified across 10 GIS samples after normalization with an average coefficient of variation is 25%. **Figure S2.** Label free versus isotope dilution peptide quantification. **(A)** The Pearson correlation with p value of normalized peak area based on with light/heavy peptide ratio of a (A) MAPT peptide SGYSSPGSPGTPGSR, (B) YWHAZ peptide, SVTEQGAELSNEER, (C) SPP1 peptide, AIPVAQDLNAPSDWDSR, (D) SPP1 peptide GDSVVYGLR, (E) VGF peptide, AYQGVAAPFPK, (F) VGF peptide NSEPQDEGELFQGVDPR and (G) STMN1 peptide SHEAEVLK. The X-axis showing the ratio of light peptide peak area divided with heavy peptide peak area, the Y-axis showing the label free measurement for the light peptide normalized peak area. **Figure S3.** Proteins reflect changes in neuronal/synaptic, glia/inflammation, myelin and metabolic group signatures in brain. The quantification showed significantly different levels in patients with early stage dementia due to AD (*n *= 37) from other non-AD neurological disease (*n *= 31) and age- and sex- matched normal controls (*n *= 20). YWHAZ, UCHL1, SYN1, MAP1B, STMN1, YWHAB, BASP1, GDA, GAP43, PACSIN1, and MAP2 proteins overlap with the neuronal/synaptic panel in brain **(A)**. MAPT, SMOC1, ITGAM, CHI3L1, CHIT1, and YWHAE proteins overlap with the glia/inflammation panel in brain **(B)**. SPP1, NEFL, NEFM, and SOD1 proteins overlap with the myelin panel in brain **(C)**. And FABP3, ALDOA, PKM, and YWHAG proteins overlap with the metabolic panel in brain **(D)**. The results are shown as the mean ± S.D. ***, ANOVA-p < 0.001, **, ANOVA-p < 0.01, *, ANOVA-p < 0.05. n.s, non-significant. **Figure S4.** Confirming identify of NEFL and NEFM peptides in CSF. (A) MS/MS spectrum of the endogenous NEFL peptide DEPPSEGEAEEEEK (m/z 787.8207, charge +2) and its corresponding isotope-labeled heavy peptide (m/z 791.8278, charge +2). The top six product ion patterns are colored. The ratio dot-product values (rdopt, 0.96) indicate the similarity of product ion pattern between endogenous peptide and heavy peptide standard. (B) MS/MS spectrum of the endogenous NEFM peptide EEGEQEEGETEAEAEGEEAEAK (m/z 1190.4751, charge +2) and its corresponding isotope-labeled heavy peptide (m/z 1194.4822, charge +2). The top six product ion patterns are colored. The ratio dot-product values (rdopt, 0.94) indicate the similarity of product ion pattern between endogenous peptide and heavy peptide standard.

## Data Availability

All raw proteomic data generated contributing to the described work will be deposited electronically at www.synapse.org/#!Synapse:syn21541022.

## Abbreviations

MS: Mass spectrometry
SRM: Selected reaction monitoring
PRM: Parallel reaction monitoring
AD: Alzheimer’s disease
CV: Coefficient of variation
LC: Liquid chromatography
CLIA: Clinical Laboratory Improvement Amendments
CSF: Cerebrospinal fluid
CAA: Chloroacetamide
TCEP: Tris-2(-carboxyethyl)-phosphine
TFA: Trifluoroacetic acid
FA: Formic acid
MCI: Mild cognitive impairment
IRB: Institutional review board
ADRC: Alzheimer’s Disease Research Center
BCA: Bicinchoninic acid assay
AGC: Automatic gain control
ELISA: Enzyme-linked immunosorbent assay
TMT: Tandem mass tag
ROC: Receiver operating characteristic
SDS-PAGE: Sodium dodecyl sulfate–polyacrylamide gel electrophoresis
