# Supplementary material for: Targeted mass spectrometry to quantify brain-derived cerebrospinal fluid biomarkers in Alzheimer’s disease
Source: Clin Proteomics. 2020 May 29;17:19. doi: 10.1186/s12014-020-09285-8 (PMC7257173; doi:10.1186/s12014-020-09285-8)
Supplement: Supplementary file 2 — Additional file 2: Figure S1. Overview of PRM targeted experiment. (A) Schematic of the workflow of the targeted PRM analyses of the replication cohort. Samples (n = 98 total) including 37 AD, 20 Controls, 31 non-AD dementia cases, and 10 pooled global internal standards (GIS) were randomized (e.g. diagnosis, age and sex) into four batches. (B) The raw and normalized peak areas (based on intensities) of reference peptides LASVSVSR, LASVSVSR, YVYVADVAAK, YVYVADVAAK, VVGGLVALR, VVGGLVALR, LLSLGAGEFK, and LLSLGAGEFK (heavy amino acids were labeled with underline) across the first and second injection of 98 samples (including 10 GIS samples). (C) The left panel shows the histogram for the coefficient of variation (CV) of all peptides quantified across the 10 GIS samples before normalization with an average coefficient of variation of 44%. The middle panel showing the coefficient of variation of all peptides quantification across 10 GIS samples after normalization with an average coefficient of variation of 27%. The right panel shows the coefficient of variation of 41 proteins quantified across 10 GIS samples after normalization with an average coefficient of variation is 25%. Figure S2. Label free versus isotope dilution peptide quantification. (A) The Pearson correlation with p value of normalized peak area based on with light/heavy peptide ratio of a (A) MAPT peptide SGYSSPGSPGTPGSR, (B) YWHAZ peptide, SVTEQGAELSNEER, (C) SPP1 peptide, AIPVAQDLNAPSDWDSR, (D) SPP1 peptide GDSVVYGLR, (E) VGF peptide, AYQGVAAPFPK, (F) VGF peptide NSEPQDEGELFQGVDPR and (G) STMN1 peptide SHEAEVLK. The X-axis showing the ratio of light peptide peak area divided with heavy peptide peak area, the Y-axis showing the label free measurement for the light peptide normalized peak area. Figure S3. Proteins reflect changes in neuronal/synaptic, glia/inflammation, myelin and metabolic group signatures in brain. The quantification showed significantly different levels in patients with early stage deme [file 12014_2020_9285_MOESM2_ESM.pdf]

Figure S1

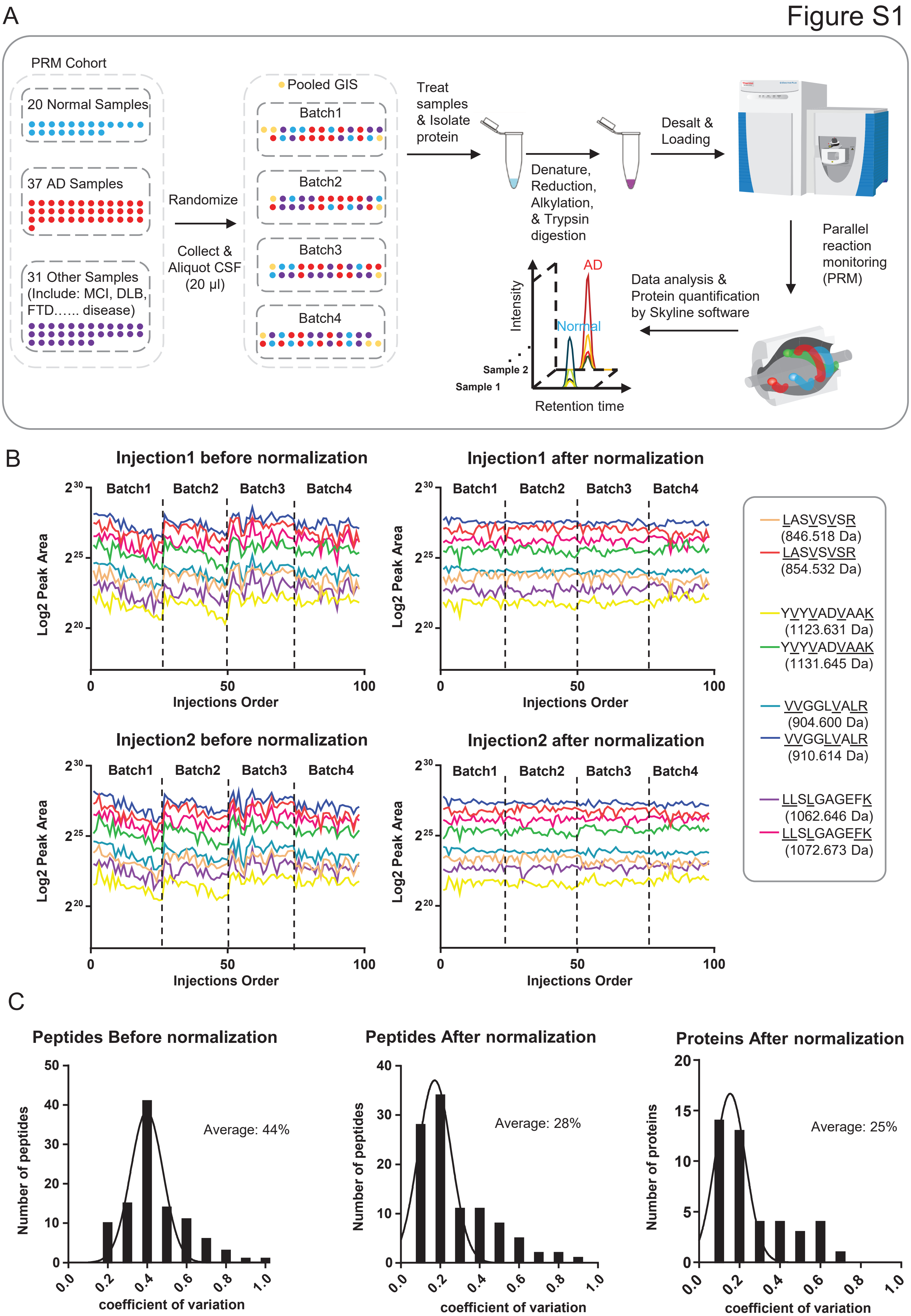
A
PRM Cohort
20 Normal Samples
37 AD Samples
31 Other Samples (Include: MCI, DLB, FTD...... disease)
Randomize
Collect & Aliquot CSF (20 µl)
Pooled GIS
Batch1
Batch2
Batch3
Batch4
Treat samples & Isolate protein
Denature, Reduction, Alkylation, & Trypsin digestion
Desalt & Loading
Parallel reaction monitoring (PRM)
Data analysis & Protein quantification by Skyline software
Intensity
AD
Normal
Sample 2
Sample 1
Retention time
B
Injection1 before normalization
Injection1 after normalization
Injection2 before normalization
Injection2 after normalization
Log2 Peak Area
Injections Order
LASVSVSR (846.518 Da)
LASVSVSR (854.532 Da)
YVYVADVAAK (1123.631 Da)
YVYVADVAAK (1131.645 Da)
VVGGLVALR (904.600 Da)
VVGGLVALR (910.614 Da)
LLSLGAGEFK (1062.646 Da)
LLSLGAGEFK (1072.673 Da)
C
Peptides Before normalization
Average: 44%
Number of peptides
Peptides After normalization
Average: 28%
Proteins After normalization
Average: 25%
Number of proteins
coefficient of variation

Figure S2

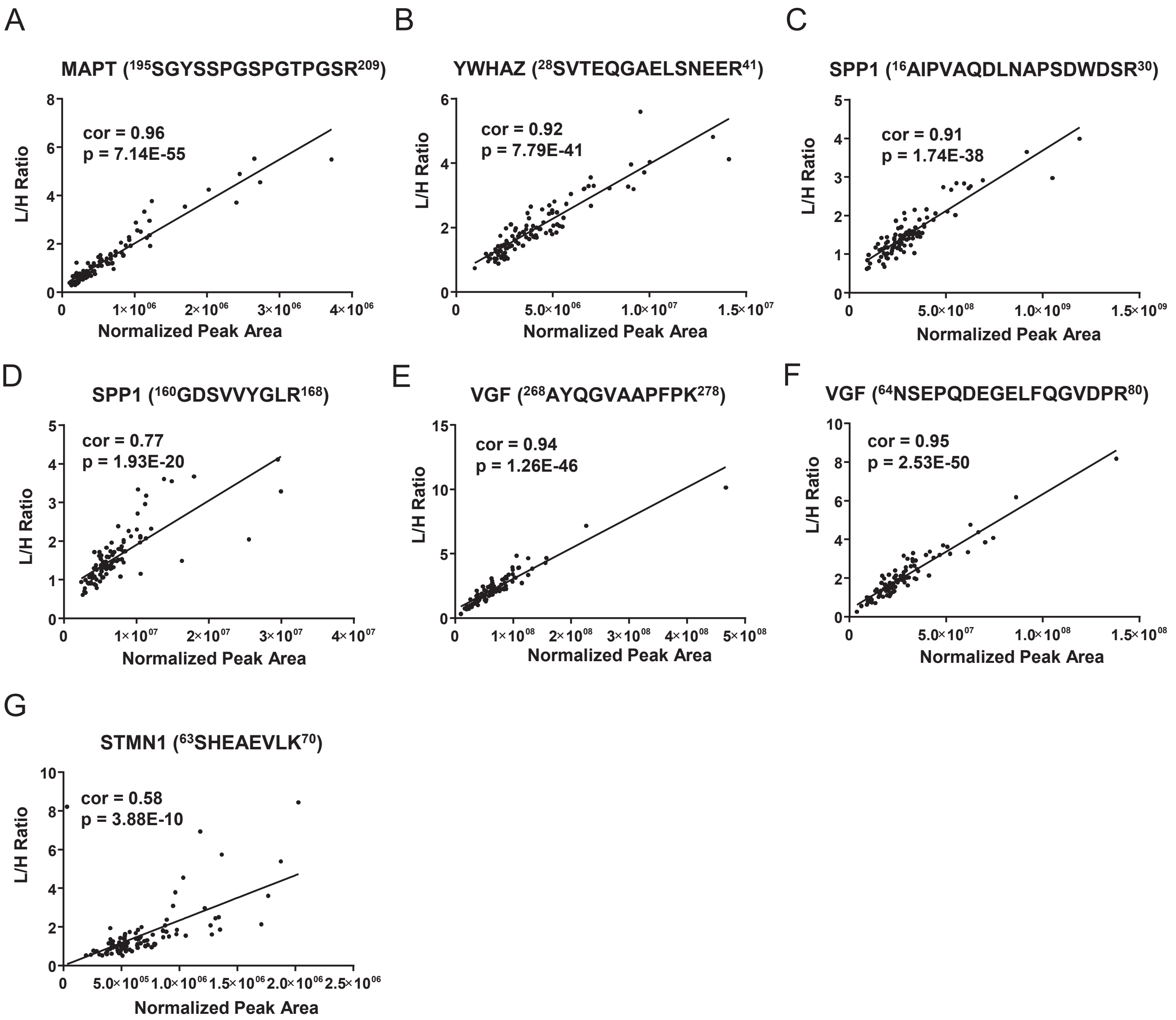

A
MAPT (^{195}SGYSSPGSPGTPGSR^{209})
cor = 0.96
p = 7.14E-55
B
YWHAZ (^{28}SVTEQGAELSNEER^{41})
cor = 0.92
p = 7.79E-41
C
SPP1 (^{16}AIPVAQDLNAPSDWDSR^{30})
cor = 0.91
p = 1.74E-38
D
SPP1 (^{160}GDSVVYGLR^{168})
cor = 0.77
p = 1.93E-20
E
VGF (^{268}AYQGVAAPFPK^{278})
cor = 0.94
p = 1.26E-46
F
VGF (^{64}NSEPQDEGELFQGVDPR^{80})
cor = 0.95
p = 2.53E-50
G
STMN1 (^{63}SHEAEVLK^{70})
cor = 0.58
p = 3.88E-10
L/H Ratio
Normalized Peak Area

Figure S3

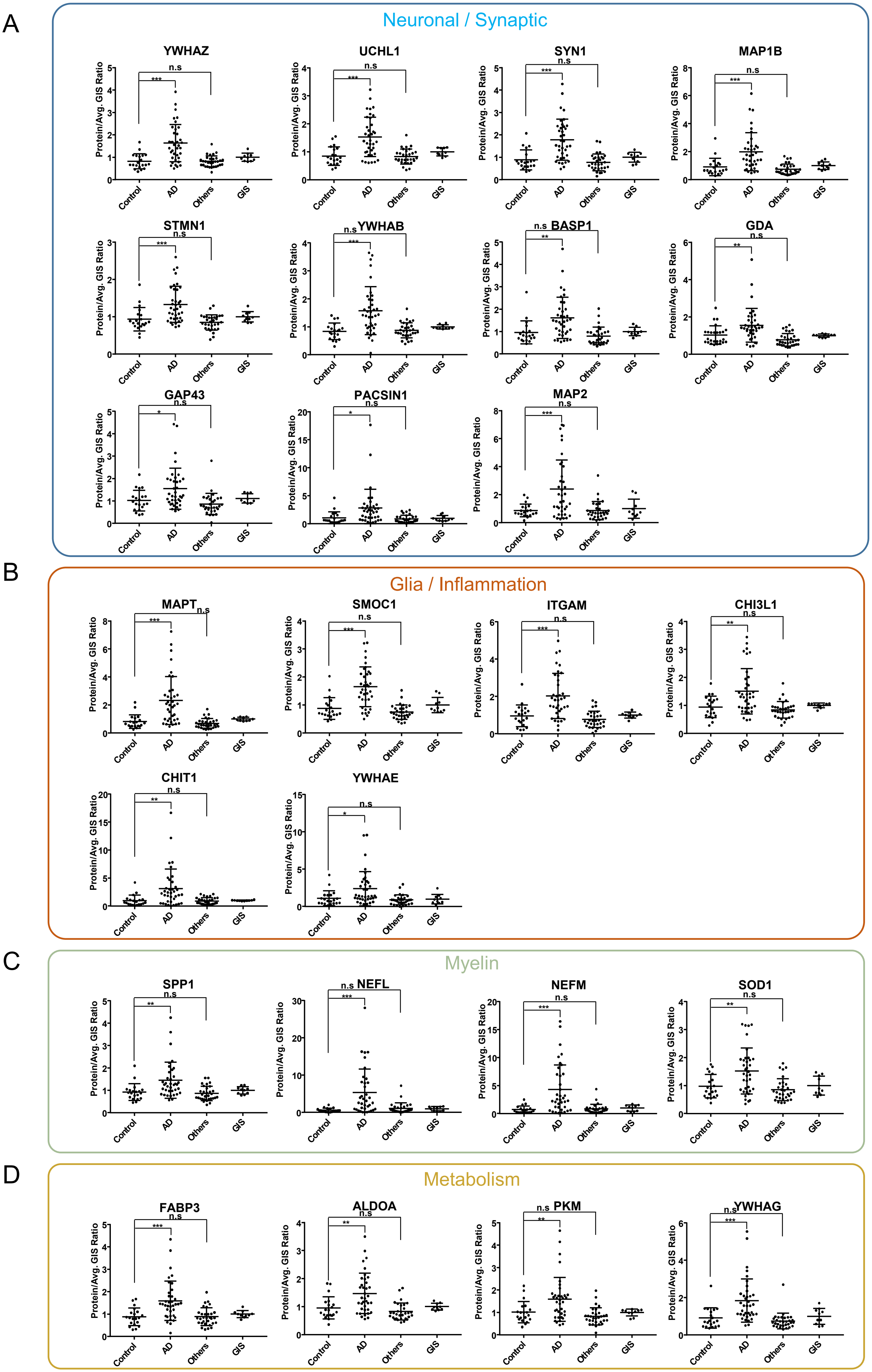

A
Neuronal / Synaptic
YWHAZ
UCHL1
SYN1
MAP1B
STMN1
YWHAB
BASP1
GDA
GAP43
PACSIN1
MAP2
B
Glia / Inflammation
MAPT
SMOC1
ITGAM
CHI3L1
CHIT1
YWHAE
C
Myelin
SPP1
NEFL
NEFM
SOD1
D
Metabolism
FABP3
ALDOA
PKM
YWHAG
Protein/Avg. GIS Ratio
Control
AD
Others
GIS
n.s
***
**
*

Figure S4

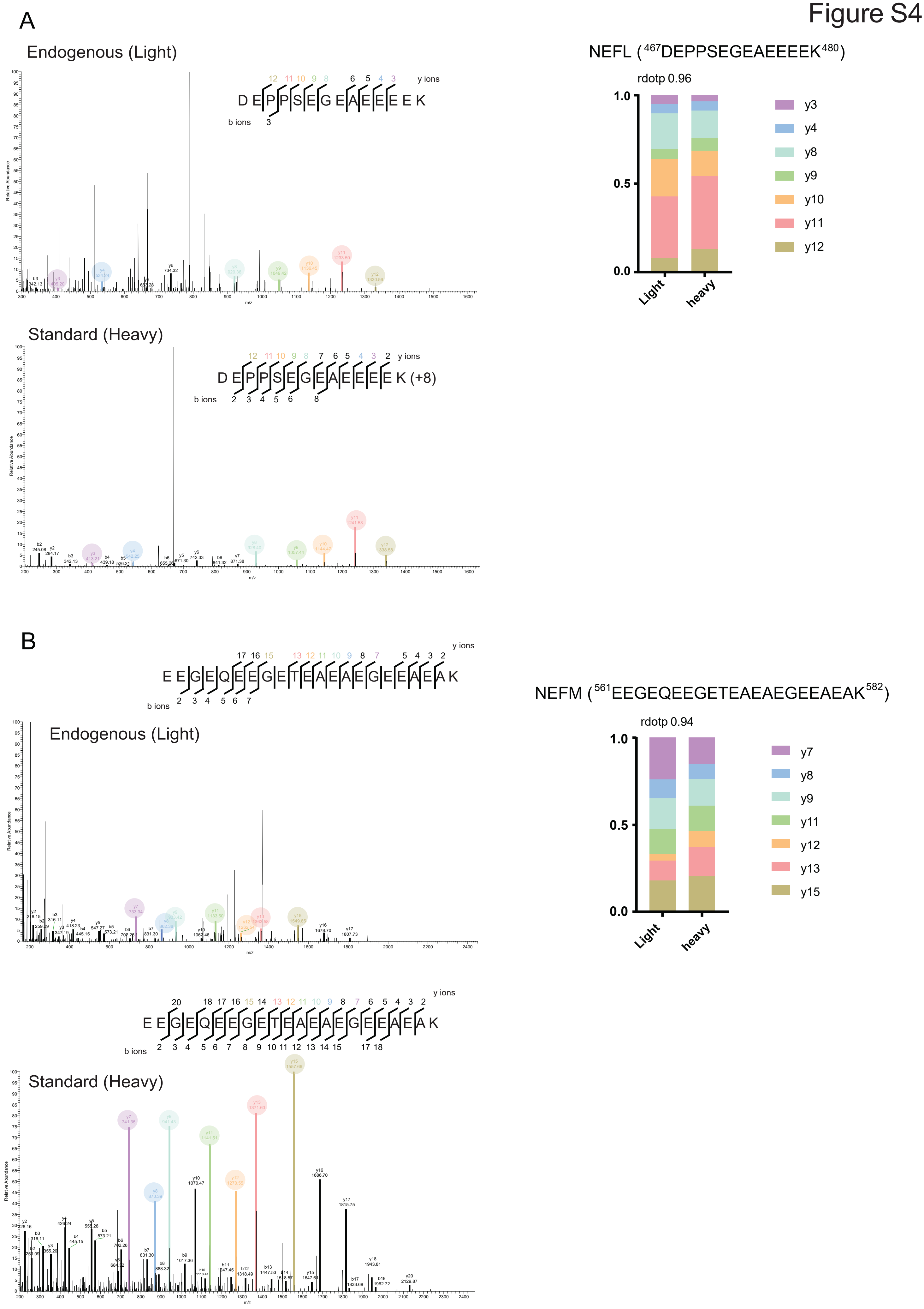
A
Endogenous (Light)
Standard (Heavy)
NEFL (467DEPPSEGEAEEEEK480)
rdotp 0.96
DEPPSEGEAEEEEK
DEPPSEGEAEEEEK (+8)
y ions
b ions
Relative Abundance
m/z
y3
y4
y8
y9
y10
y11
y12
Light
heavy
B
NEFM (561EEGEQEEGETEAEAEGEEAEAK582)
rdotp 0.94
EEGEQEEGETEAEAEGEEAEAK
y7
y8
y9
y11
y12
y13
y15
